# SARS Antibody Testing in Children: Development of Oral Fluid Assays for IgG Measurements

**DOI:** 10.1128/spectrum.00786-21

**Published:** 2022-01-05

**Authors:** Katja Hoschler, Samreen Ijaz, Nick Andrews, Sammy Ho, Steve Dicks, Keerthana Jegatheesan, John Poh, Lenesha Warrener, Thivya Kankeyan, Frances Baawuah, Joanne Beckmann, Ifeanichukwu O. Okike, Shazaad Ahmad, Joanna Garstang, Andrew J. Brent, Bernadette Brent, Felicity Aiano, Kevin E. Brown, Mary E. Ramsay, David Brown, John V. Parry, Shamez N. Ladhani, Maria Zambon

**Affiliations:** a Virus Reference Department, Public Health England, London, United Kingdom; b Immunisation and Countermeasures Division, Public Health England, London, United Kingdom; c Microbiology Services Laboratory, NHS Blood and Transplant, Bristol, United Kingdom; d East London NHS Foundation Trust, London, United Kingdom; e Derbyshire Healthcare NHS Foundation Trust, Derby, United Kingdom; f Manchester University NHS Foundation Trust, Manchester, United Kingdom; g Birmingham Community Healthcare NHS Trust, Aston, United Kingdom; h Oxford University Hospitals NHS Foundation Trust, Oxford, United Kingdom; i University of Oxford, Oxford, United Kingdom; j Fundação Oswaldo Cruz-Fiocruz, Instituto Oswaldo Cruz, Laboratório de Vírus Respiratórios e do Sarampo, Rio de Janeiro, Rio de Janeiro, Brasil; k Paediatric Infectious Diseases Research Group, St. George’s University of London, London, United Kingdom; Quest Diagnostics Nichols Institute

**Keywords:** antibody, COVID-19, schools, surveys, children, oral fluid

## Abstract

Seroepidemiological studies to monitor antibody kinetics are important for assessing the extent and spread of SARS-CoV-2 in a population. Noninvasive sampling methods are advantageous for reducing the need for venipuncture, which may be a barrier to investigations, particularly in pediatric populations. Oral fluids are obtained by gingiva-crevicular sampling from children and adults and are very well accepted. Enzyme immunoassays (EIAs) based on these samples have acceptable sensitivity and specificity compared to conventional serum-based antibody EIAs and are suitable for population-based surveillance. We describe the development and evaluation of SARS-CoV-2 IgG EIAs using SARS-CoV-2 viral nucleoprotein (NP) and spike (S) proteins in IgG isotype capture format and an indirect receptor-binding-domain (RBD) IgG EIA, intended for use in children as a primary endpoint. All three assays were assessed using a panel of 1,999 paired serum and oral fluids from children and adults participating in school SARS-CoV-2 surveillance studies during and after the first and second pandemic wave in the United Kingdom. The anti-NP IgG capture assay was the best candidate, with an overall sensitivity of 75% (95% confidence interval [CI]: 71 to 79%) and specificity of 99% (95% CI: 78 to 99%) compared with paired serum antibodies. Sensitivity observed in children (80%, 95% CI: 71 to 88%) was higher than that in adults (67%, CI: 60% to 74%). Oral fluid assays (OF) using spike protein and RBD antigens were also 99% specific and achieved reasonable but lower sensitivity in the target population (78%, 95% CI [68% to 86%] and 53%, 95% CI [43% to 64%], respectively).

**IMPORTANCE** We report on the first large-scale assessment of the suitability of oral fluids for detection of SARS-CoV-2 antibody obtained from healthy children attending school. The sample type (gingiva-crevicular fluid, which is a transudate of blood but is not saliva) can be self collected. Although detection of antibodies in oral fluids is less sensitive than that in blood, our study suggests an optimal format for operational use. The laboratory methods we have developed can reliably measure antibodies in children, who are able to take their own samples. Our findings are of immediate practical relevance for use in large-scale seroprevalence studies designed to measure exposure to infection, as they typically require venipuncture. Overall, our data indicate that OF assays based on the detection of SARS-CoV-2 antibodies are a tool suitable for population-based seroepidemiology studies in children and highly acceptable in children and adults, as venipuncture is no longer necessary.

## INTRODUCTION

SARS-CoV-2 virus causes coronavirus disease 2019 (COVID-19), which is primarily a self-limiting upper respiratory illness but can be severe and fatal, especially in older adults (1, 2). Asymptomatic, mild, and subclinical infection is common, particularly in children and adolescents (3). Testing only symptomatic individuals misses a significant proportion of cases and, therefore, underestimates the scale and spread of infection, which is information critical for understanding transmission. The presence of SARS-CoV-2 antibodies provides a more robust measure of prior infection, irrespective of symptom status. Large-scale seroepidemiological programs provide crucial evidence in the monitoring of the progress of the pandemic and the impact of control measures.

The scale of SARS-CoV-2 infection in children and young people is uncertain, and their role in infection and transmission remains unclear (4, 5). Seroepidemiological programs based on testing of residual blood donations and clinical microbiology samples have yielded early insights into progress of the pandemic in England in adults (6, 7), but an important barrier for such programs, particularly in younger adults and children, is the availability of large numbers of representative blood samples.

The use of oral fluid (OF) for infection surveillance was pioneered in the United Kingdom, where it has been successfully used across a range of pathogens for several decades and to support the evaluation of the childhood vaccine program (8–12). OF is a complex body fluid (13, 14) derived from different anatomical sources, comprising saliva, which is a glandular secretion of aqueous fluid enriched in enzymes and electrolytes and secretory IgA, and gingiva-crevicular fluids from the capillary beds at the margin between teeth and gumline. The latter fluids are a transudate from serum, containing IgG and IgM at approximately 1/800 and 1/400, respectively, of that which is found in serum (8). Gingiva-crevicular fluids are preferred for serological assay development due to the higher concentration of IgG and are selectively sampled through the use of collecting devices, such as Oracol (15), which are used to brush the gumline and stimulate the transudation of fluid while minimizing the saliva content. Collection of oral fluids is suited to sampling populations such as children and underserved groups because it does not require the use of venipuncture. The specimen can be self collected, guided by videos and pictorial instructions (8).

Public Health England (PHE), an Executive Agency of the UK Department of Health, initiated SARS-CoV-2 surveillance in primary schools across England, sKIDs (5, 16). In total, 131 schools across England were recruited; 86 schools provided weekly nasal swabs for SARS-CoV-2 reverse transcriptase PCR (RT-PCR) and 45 schools provided a blood sample, a nasal swab sample, and an oral fluid sample at the beginning and end of the autumn term in 2020 (5), providing population-based materials to assess the feasibility and performance of oral fluid tests.

At the time of initiation of this study, testing of specimens other than serum or plasma for antibody was very limited, commercial assays had not been widely tested with specimens such as OF, SARS-CoV-2 surveillance data in children was scarce and urgently needed to make appropriate public health decisions, and shortages in laboratory tests and consumables were observed and anticipated globally. Taken together, this led to our decision to design an in-house solution. In this study, we evaluated three different in-house enzyme immunoassays for SARS-CoV-2 antibodies in OF against paired blood samples taken from children participating in a national surveillance program with the primary objective to develop a serology test for use in children to simplify population studies and provide support for national seroprevalence studies in this age group.

## RESULTS

**FIG 1:**
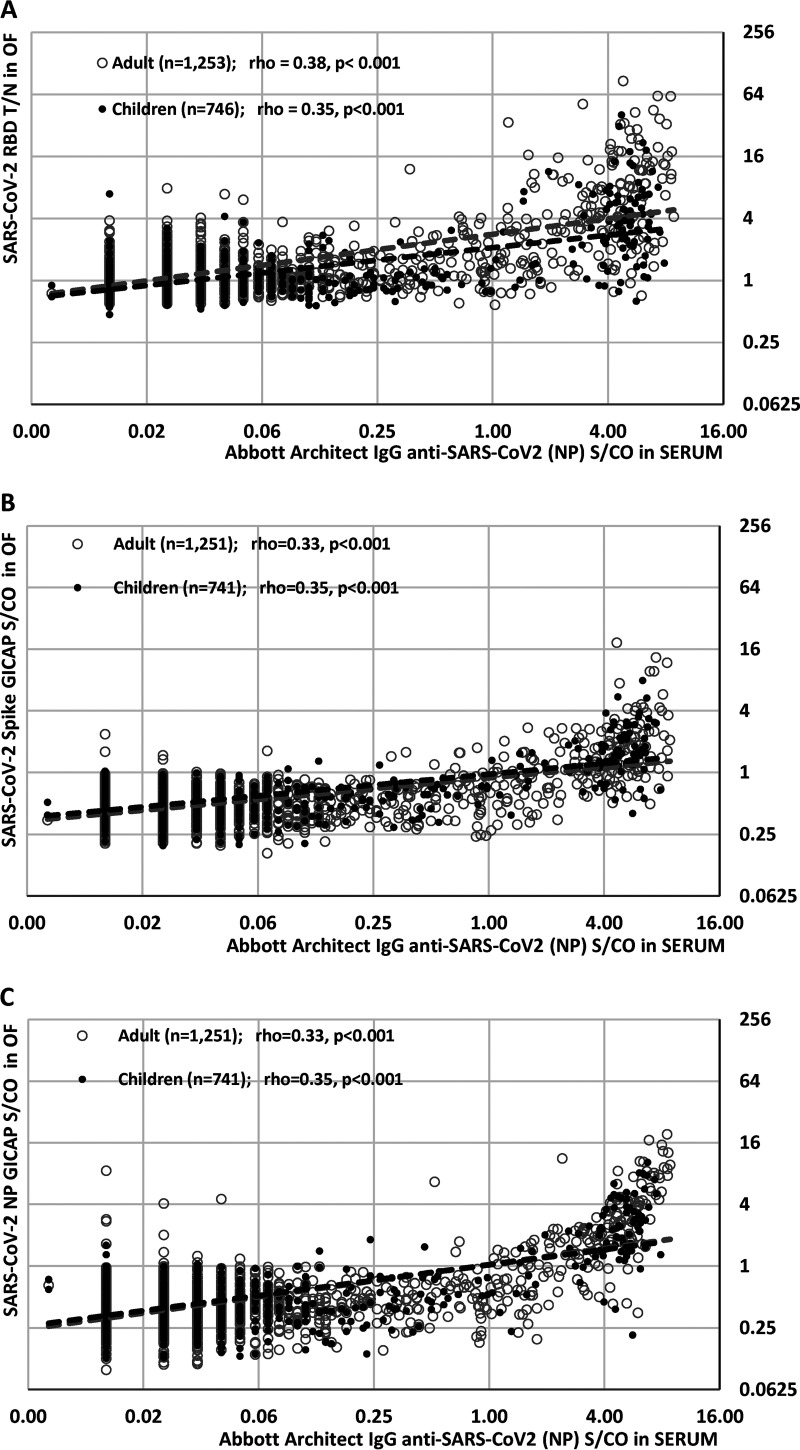
Scattergrams of Abbot Architect IgG anti-SARS-CoV-2 S/CO determined in sera versus test result determined from concomitantly collected and paired oral fluids analyzed in (A) IgG anti-SARS-CoV-2 (RBD, indirect format), (B) IgG anti-SARS-CoV-2 (spike, capture format), and (C) IgG anti-SARS-CoV-2 (NP, capture format). All data are log transformed. Data from children are shown in solid black dots, and samples from staff (adults) are shown in gray circles; numbers are shown in graph. Dashed lines represent data trends in each assay, with cutoffs for OF EIAs and Abbot Architect indicated by vertical and horizontal dotted lines, respectively. Spearman’s rho and *P* value given for each trend. T/N, test to negative ratio; S/CO, signal to cutoff ratio.

Paired serum and OF samples from 1,999 subjects were tested (Table S1). SARS-CoV-2 antibody seropositivity was confirmed in 12% (92/746) of children and 16% (196/1,253) of staff from blood samples taken during the period of May to July 2020, which is comparable to age-matched antibody seroprevalence in the local community at the time (5). All OF specimens were assayed for total IgG concentration to account for variations in this analyte arising from variations in the self-sampling technique. The overall distribution of total IgG in OF collected from students and staff indicated that 20/1,999 (1.0%) OF specimens had undetectable IgG titers and a further 67/1,999 (3.3%) had an IgG concentration of >0.1 mg/L and <1.0 mg/L. Thus, 95.7% (1,913/1,999) had an IgG concentration of ≥1 mg/L, with the vast majority (86.8% [1,736/1,999]) with a concentration of ≥2.0 mg/L. Tobit analysis of logged total IgG (in OF) with censoring of IgG concentrations at 1 and 15 mg/L and with adjustment for adult/children showed that the concentration in those that were positive (1.32-fold, 95% CI [1.13 to 1.55]) in the serum assay was slightly higher than that in those that were negative, but this association was not strong (Fig. 1). Children were more likely than adults to provide an OF with no detectable (14/746 [1.9%] versus 6/1,253 [0.5%]) or low (0.2 to 1 mg/L) IgG antibody titers (57/746 [7.6%] versus 10/1,253 [0.8%]).

### Performance of three OF EIAs.

**FIG 2:**
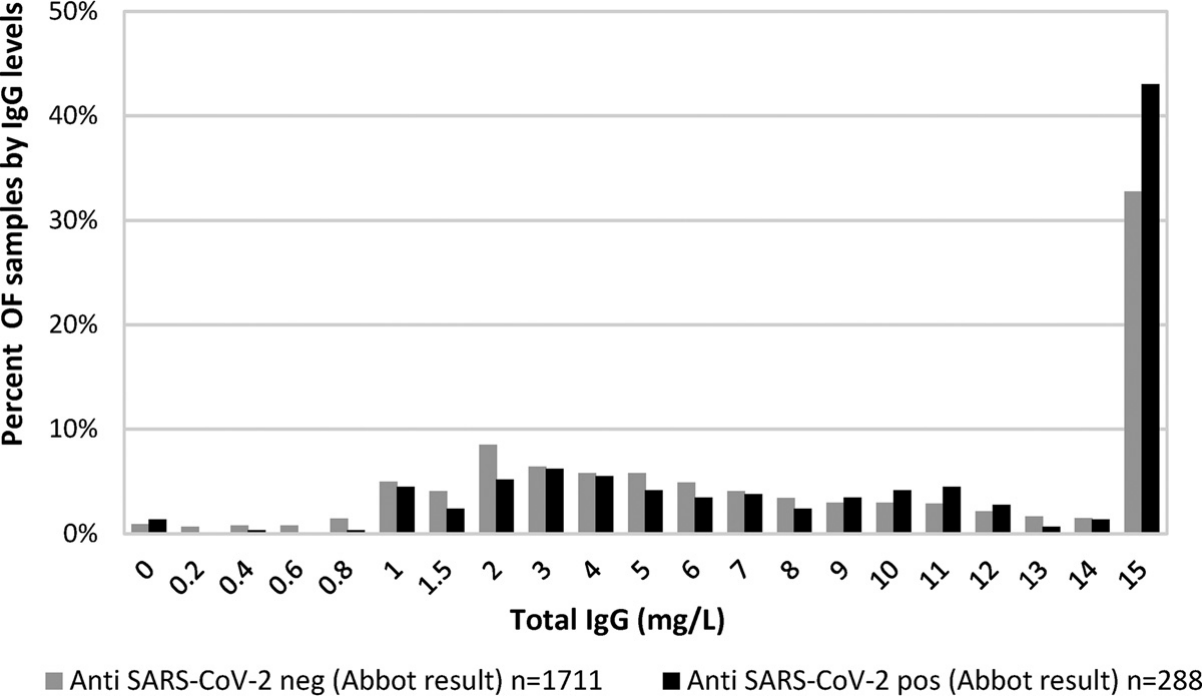
Distribution of total IgG in OF by SARS-CoV-2 serum result (from Abbot Architect analyzer). Samples were grouped into those above and below seropositivity cutoff in the Abbot Architect IgG anti-SARS-CoV-2 (S/CO of <0.8 and ≥0.8, respectively).

Comparison of serum with oral fluid IgG antibody titers using each of the three OF assays showed a strong and statistically significant quantitative correlation between the serum IgG signal/cutoff (S/CO) ratio and each of the oral fluid EIA antibody titers (Fig. 2). The number of discordant results, with antibody detectable in blood but not in OF, was most evident with the receptor-binding domain (RBD) EIA and least evident with the anti-nucleoprotein (anti-NP) IgG capture EIA (GICAP). The sensitivities of the GICAP EIAs (NP, 80% and spike, 78%) were significantly better than those of the indirect EIA (RBD: 53%) when OFs from children were tested, while specificities were similar, at 99% (Table 1 and Fig. S1). Receiver operating characteristic (ROC) curve analysis confirmed the superiority of the NP and spike (S) GICAP EIAs over the indirect RBD EIA in the correct classification of oral fluids from children, with the area under the curve (AUC) for both assays statistically larger than that for the RBD EIA. For OFs from adults (staff), sensitivities were overall lower than those for OFs from children, and the AUC were comparable, but the NP GICAP EIA was the most sensitive and offered marginally better specificity than the S GICAP and the RBD EIA.

**TABLE 1:** Sensitivity and specificity findings for the 3 oral fluid IgG anti-SARS-CoV-2 EIAs at their optimal cutoff based on status based on a serum test in the Abbott Architect SARS-CoV-2 IgG assay^*a*^

EIA	Cutoff	Sensitivity		Specificity	
		95% CI	*n*/N	95% CI	*n*/N
Children (*n* = 746)					
RBD indirect	3.0	53% (43%–64%)	49/92	99% (98%–100%)	649/654
Spike capture	1.0	78% (68%–86%)	71/91^*b*^	99% (98%–100%)	645/650^*c*^
Nucleoprotein capture	1.0	80% (71%–80%)	73/91^*b*^	99% (98%–100%)	644/650^*c*^
Staff (*n* = 1,253)					
RBD indirect	3.0	60% (53%–67%)	117/196	98% (97%–99%)	1,035/1,057
Spike capture	1.0	58% (51%–65%)	113/195^*d*^	99% (98%–99%)	1,044/1,056^*e*^
Nucleoprotein capture	1.0	67% (60%–74%)	131/195^*d*^	99% (98%–99%)	1,041/1,056^*e*^

a CI, confidence interval (set at 95%); n, number (in a category); N, total number (of individuals).

b,c,d A single specimen from each of these categories was of insufficient volume to permit testing by the 2 IgG capture assays.

e Four specimens from this category were of insufficient volume to permit testing by the 2 IgG capture assays.

### Utility of testing each oral fluid specimen for total IgG content.

**FIG 3:**
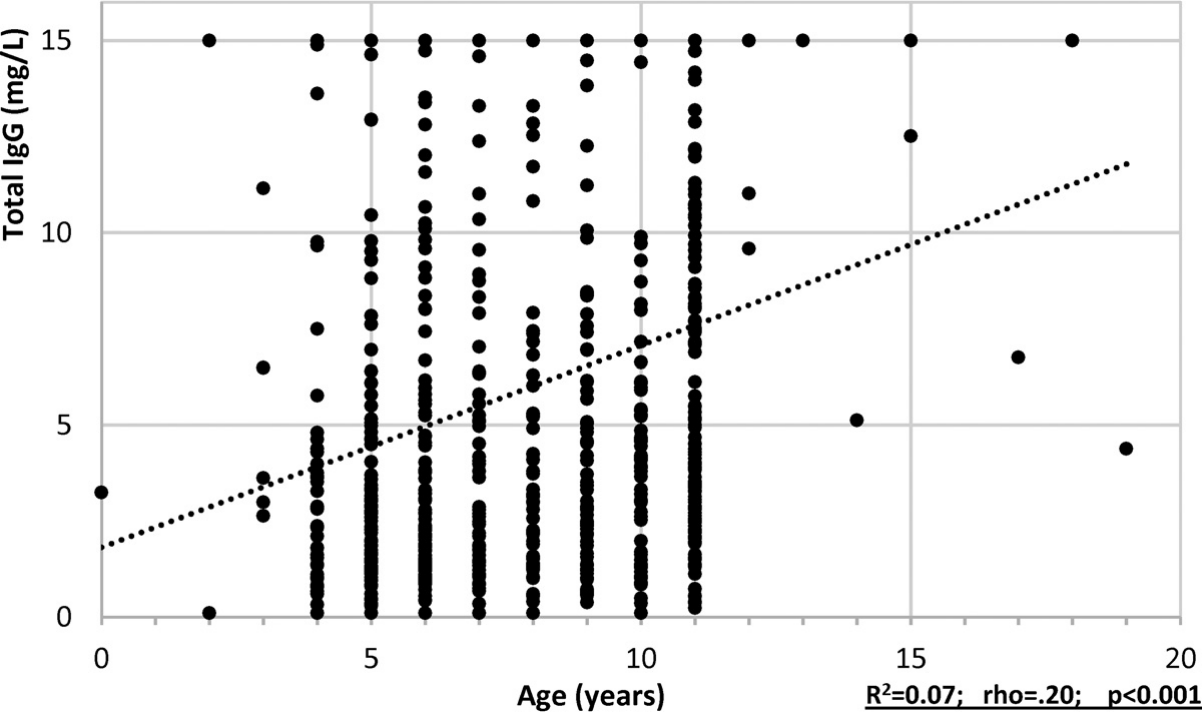
Scattergram of total IgG measured in oral fluid by age of subject (children only; 708 children with known age included). Dotted line indicates trend, with Spearman’s rho and *P* value as well as R^2^.

**FIG 4:**
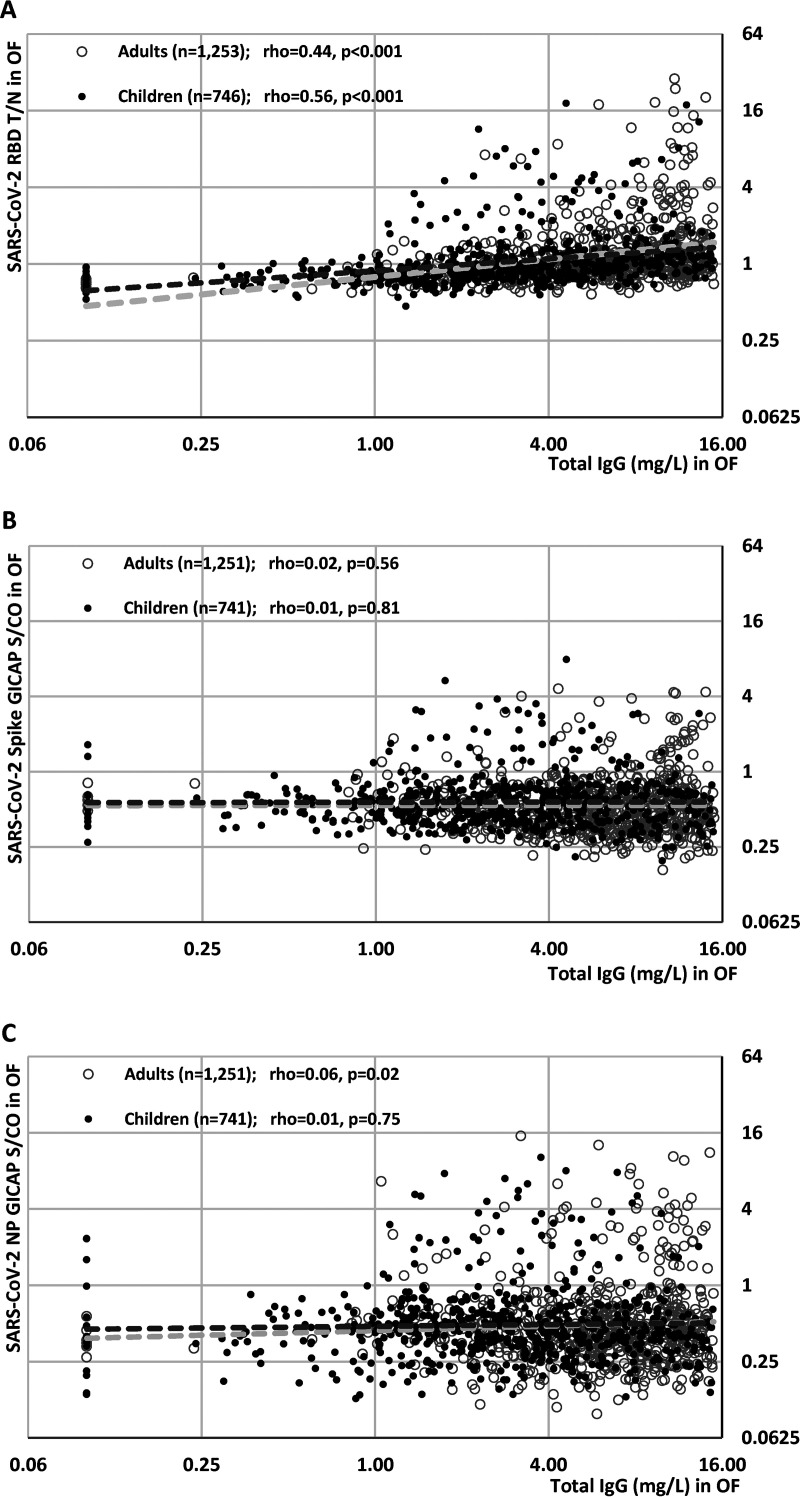
Scattergrams of total IgG concentration (in mg/L) determined in oral fluids versus test result determined in (A) IgG anti-SARS-CoV-2 (RBD, indirect format), (B) IgG anti-SARS-CoV-2 (spike, capture format), and (C) IgG anti-SARS-CoV-2 (NP, capture format). Data from children are shown in solid black dots and samples from staff (adults) are shown in gray circles. All data are log transformed. The upper limit of quantification in the total IgG determination is 15 mg/mL; data points from samples with IgG of >15 mg/L were excluded from graphs. Number of samples within this limit: children, *n* = 619; number of samples from adults/staff, N = 695. Dashed lines represent data trends in each assay, with the cutoff for the OF EIAs indicated by dotted lines. Spearman’s rho and *P* value are given for each trend. T/N, test to negative ratio; S/CO, signal to cutoff ratio.

Total IgG concentrations in Oracol OF samples taken increased with age (Fig. 3 and see Fig. S2 in the supplemental material). Overall, there was poor correlation between total IgG concentrations in oral fluids and SARS-CoV-2 IgG antibody titers for each of the three OF EIAs, although there was a declining trend in RBD EIA IgG titers associated with declining total IgG concentration for both children and staff (Fig. 4). For the GICAP EIAs, such a trend was absent for OF samples from children, although there was a modest declining trend in SARS-CoV-2 IgG antibody titers as total IgG concentrations fell, but it was much less pronounced than that observed with the RBD EIA. When OF anti-NP EIA sensitivity was stratified based on total IgG concentration, the overall sensitivity remained unchanged until total IgG concentration fell to <1 mg/L (Fig. 4). While OF results in children maintained high sensitivity at all IgG concentrations of >1 mg/L, sensitivity in adults declined as total IgG fell.

It was not possible to identify a clear boundary at which total IgG was associated with loss of sensitivity for SARS-CoV-2 NP IgG detection. An arbitrary total IgG threshold of 0.2 mg/L, or even 1 mg/L, would exclude only 1% or 4.3% of negative specimens, respectively, from prevalence estimates. This would lead to very small changes in measured prevalence; for example, a seroprevalence of 20% would change to 20.2% or 20.7%, respectively.

## DISCUSSION

This study forms part of a national program of SARS-CoV-2 infection and transmission studies in primary schools whereby paired samples were taken from students and staff to estimate acute infection and seroprevalence over a 6-month period. As part of the study, contemporaneous OF samples were taken from asymptomatic students and staff to develop and validate a noninvasive alternative to blood sampling for seroepidemiological surveillance applications and for use in outbreak investigations. Our results show that SARS-CoV-2 antigen-specific antibody responses in oral fluids reflect those observed in serum and that SARS-CoV-2 antigen-specific IgG can be used to detect SARS-CoV-2-seropositive individuals for the purpose of seroepidemiology. Taking into consideration the three OF assays and the reproducibility, robustness, reagent supply chain, and sustainability of service delivery, the IgG isotype-specific SARS-CoV-2 NP capture EIA has been adopted as the principal test for the OF-based SARS-CoV-2 antibody surveillance studies in children performed and organized by PHE (17, 18). The observed sensitivity and specificity (80% and 99%, measured against the standard assay used in the serum-based surveillance) make our candidate test a suitable tool for COVID-19 surveillance in children.

Several viral antigens were evaluated. Viral NP and S proteins are used most frequently in commercial immunoassays (19). Each virion carries on average more than 2,000 copies of the nonglycosylated NP protein, and some data suggest that SARS-CoV-2 NP antibody detection may be more sensitive than S protein antibody detection for detecting early infection (20). The NP of SARS-CoV-2 shows between 58% and 65% similarity to NP of seasonal coronaviruses, and cross-reactivity has been observed in multiple studies (21, 22). The S protein, specifically the RBD region of this protein, is the target for neutralizing antibodies, and antibodies against the S1 subunit of the spike protein or against RBD have been observed to correlate with neutralizing antibody, although not all spike/RBD antibodies detected in binding assays confer neutralization (23).

For OF sampling, the IgG content of the extracted specimen represents, on average, a dilution of roughly 1/1,000 of that in blood plasma (24, 25). As a result, antibody tests using OFs tend to have lower sensitivity than those designed for serum, but this analyte is successfully used in seroepidemiology studies for other viruses, e.g., measles and HIV (26–28). The concentration of OFs varies between individual specimens, ranging from <0.5 mg/L to >30 mg/L (29). This variability in IgG concentrations makes the choice of assay format more critical. Generally, GICAP antibody tests have proved to be most robust when detecting viral antibodies in OFs, as they are tolerant to the very wide range of IgG concentration. The strength of any reactivity in a capture assay is dependent on the proportion of total IgG that is specific for the target antigen, in this case, the SARS-CoV-2 viral proteins. Consequently, if there is sufficient total IgG to saturate the anti-IgG binding sites on the solid phase, the quantity of captured antibodies, while only a part of the total IgG captured, will be constant. In our study, measurement of total IgG content to assess OF sample quality revealed a strong correlation with RBD EIA reactivity but not with NP or S protein EIAs. This reflects the greater robustness of the IgG isotype capture EIAs when testing clinical samples, including OFs, that have highly variable total IgG content. This observation provides some insight into the differences in anti-SARS-CoV-2 antibody sensitivity between the different OF assays assessed. We also confirmed lower OF IgG concentrations in young children, which increase with age, reflecting plasma IgG levels which also increase with age, and this probably explains why the RBD EIA sensitivity was lower in children than in staff. Other reasons for lower IgG content in children’s oral fluids may be physiological or, in some cases, failure to follow instructions, such as not collecting an OF sample for the recommended 2 min. Our own previous published work has shown that standardization using the total IgG concentration in oral fluid can improve correlation of results from an oral fluid EIA and a gold standard assay performed on sera (30). In the context of serology for SARS-CoV-2, it has been published that the IgG/IgM/IgA content in saliva can be used to normalize the specific antibody measured (31). Overall, however, the low number of inadequate samples (<2%) and the additional costs and demands on laboratory time and facilities associated with measuring total IgG for every OF sample far outweigh the marginal gain of confirming the quality of the sample for measuring SARS-CoV-2 antibodies when there is very little impact on assay performance as illustrated in Table 2 for the NP GICAP, the assay chosen for the sKIDs surveillance.

**TABLE 2:** Sensitivity of GISAC EIA for detection of IgG anti-SARS-CoV-2 NP antibody in oral fluid specimens by total IgG concentration^*a*^

IgG conc.	Seropositive children			Seropositive staff			Seropositive overall		
	No. NP positive	No. NP negative	% positive	No. NP positive	No. NP negative	% positive	No. NP positive	No. NP negative	% positive
>10 mg/L	22	7	75.9%	94	39	70.7%	116	46	71.6%
2–10 mg/L	37	7	84.1%	34	20	63.0%	71	27	72.4%
1–2 mg/L	12	2	85.7%	3	3	50.0%	15	5	75.0%
<1mg/L	2	2	50.0%	0	2	0.0%	2	4	33.3%
									
Overall	73	18	80.2%	131	64	67.2%	204	82	71.3%

a IgG, immunoglobulin subclass G (total, non-SARS-CoV-2-specific IgG).

As anticipated, overall sensitivity of detection of SARS-CoV-2 IgG OF antibodies was lower than that of serum antibodies. By comparing three different OF SARS-CoV-2 antibody assays, we found that the sensitivities of the IgG isotype capture EIAs (80% for NP, 78% for S) were significantly higher than those of the indirect EIA (RBD, 53%) when testing OFs from children, while specificities were similar at 99% for all three assays. Overall, the observed sensitivity and specificity data is in keeping with those of other recent studies (31–34). The comparison between the two capture assays shows that the assay using NP antigen was more sensitive than the S based assay which is consistent with some previous findings in sera and plasma (20, 35), but differs from observations in a recent study comparing the results from sera and saliva collected from children seeking medical care, analyzed using an exploratory Luminex assay including S, RBD, and NP antigens (36). While in a multiplex magnetic microparticle assay using multiple SARS-CoV-2 antigens, the performance with NP-based antigens for OFs from adults was consistently more sensitive than that with spike-based assays (34), the comparison of antibody levels in serum and saliva of Dutch children showed higher IgG levels to S than to NP, with higher levels in saliva than in serum. The difference of the latter result from ours and those of other studies may be partly explained in the difference in specimen type, saliva as collected by Keuning et al. versus the oral fluid we analyzed (36). It has been noted that in adults, up to 20% of seronegative individuals had mucosal SARS-CoV-2 S-specific antibodies, some of which showed *in vitro* neutralizing capacity (37), and the authors suggested that S protein-specific IgA and IgG at mucosal sites, even in the absence of systemic responses, may be associated with mild COVID-19 and also with younger age.

We used serum SARS-CoV-2 nucleoprotein antibody positivity to infer prior infection in participants because PCR availability was very limited in the community at the time of recruitment. In our cohort of primary school students and staff, recruited during the first 2 weeks of June 2020, 300 of 2,197 participants (13.7%; 95% CI, 10.8 to 16.9) had evidence of prior infection by testing positive for serum SARS-CoV-2 nucleoprotein antibodies: 91/816 students (1.2%; 95% CI, 7.9 to 15.1) and 209/1,381 staff members (15.1%; 95% CI, 11.9 to 18.9), which were also comparable to local community seroprevalence rates in adults, but none of these had PCR-confirmed infections. Moreover, after initiation of the study, few PCR positives (3 in ∼40,000 tests over 6 weeks) were found within the weekly PCR testing arm of the sKIDs study, as infection rates were low in children during this time and thus insufficient to assess sensitivity or specificity of our OF assays (5). The choice of PCR detection as a reference comparator to assign infection status, rather than serum measurements of the same antibody as recently described by Pisanic et al. (34), will underestimate the number of infections, thereby providing an underestimate of serological prevalence and inflating the assumed sensitivity of the OF assay. A reference classification using a molecular detection assay and a serum antibody assay in combination and excluding samples from antibody-negative, PCR-positive individuals is expected to improve sensitivity and specificity of an assessed OF assay, as demonstrated in reference 38.

To overcome these limitations, we chose a commercial serum antibody assay that had been assessed by our agency, with high sensitivity and specificity, using more than 160 serum samples from PCR-confirmed individuals taken at various time points postinfection and more than 1,100 negative samples (prepandemic/baseline samples) (39) as “gold standard assay.” Although antibody waning has been observed >3 months postinfection with this Abbott SARS-CoV-2 IgG assay (detecting nucleoprotein antibody), which has not been noted to the same extent with other assays such as Roche N or with spike protein antibody platforms (5, 40), the impact on the overall study findings is limited, because participants had blood and OF samples taken within a few weeks (June 2020) after their primary infection (March to April 2020) and assay-specific waning would not yet have substantially reduced the antibody levels in the partaking individuals.

Another limitation is that we focused on the measurement of circulating IgG and did not measure secreted antibody, consistent with our primary objective. Most of the seroprevalence work performed by PHE focuses on measurement of specific IgG, as it is known to be a reliable indicator of previous exposure and / or infection. While IgM and IgA have been demonstrated to be present in blood after SARS-CoV-2 (41), both have been shown to be transient and thus are not good analytes for seroprevalence work. Moreover, little is known about the interpretation of systemic IgA antibody levels for seroprevalence purposes; while secreted IgA has been hypothesized to prevent symptomatic SARS-CoV-2 infection (37), the presence of very high serum IgA levels has been linked with severe infections and age in adult patients (42), and severe infections are unlikely in our young cohort. On the other hand, the heterogeneity in the reactivity of IgG and IgA antibodies for S, RBD, and N antigens observed in saliva from Dutch children (36) illustrates that salivary IgA results are difficult to interpret and require careful consideration with respect to antigen and assay format.

Finally, the limitation of using a single antigen assay is that antibody kinetics may vary over time. Following mild SARS-CoV-2 infection, for example, serum nucleoprotein antibodies decline more rapidly than spike protein antibodies (21), but this may differ for severe illness and may also be dependent on the assays used (43). Additionally, little is known about antibody kinetics in children, which may differ over time since infection. It is, therefore, possible that exclusive use of the NP capture assay may need to be reevaluated in future, when antibody from naturally acquired infection starts to wane. Since current vaccines induce spike protein antibodies, inclusion of a spike protein antibody assay would allow distinction between antibodies induced following natural infection and those induced following vaccination, especially in the context of discussions around vaccination of children. We will continue to evaluate and reassess the selected assay with longitudinal oral fluids and serum samples collected from children and adults with confirmed SARS-CoV-2 infection. Furthermore, with the recent FDA approval and CE marking of the first commercial tests for antibody in saliva (CovAB COVID-19 saliva antibody test by Diabetomics, USA or rapid saliva protein test by Medusa-19 Ltd, UK), we may be in a position to evaluate these and assess their suitability for the analysis of OFs from sKIDs or other surveillance programs. The progress made in LUMINEX technology and the benefit of reduced use of sample volume and synchronized analysis of multiple antigens also make this an attractive platform worth investigating in the future.

## MATERIALS AND METHODS

### Ethics statement.

The work described here falls outside the Health Research Authority remit for ethical review, in accordance with the revised guidance in the Governance Arrangements for Research Ethics Committees (GAfREC) that was released in September 2011. The surveillance protocol has been subject to an internal ethical review by the PHE Research Ethics and Governance Group to ensure that it is fully compliant with all regulatory requirements and was approved by the Public Health England Research Ethics Governance Group (R&D REGG ref: NR0209, May 2020) (16).

### Recruitment and sample collection.

Headteachers in participating primary schools sent the study information pack to parents and staff at the start of the study, and those interested in taking part were asked to sign a consent form and complete a short questionnaire. Parents provided written informed consent for their children to participate. PHE investigators attended the school premises in the period of 28 May to 10 July 2020, took nasal swabs and blood samples from participating children, and provided guidance and supervision of the oral fluid self sampling (Table S1; for study protocol, see reference 16).

All individuals sampled were attending school and were asymptomatic at the time of sampling. Otherwise, there were no additional exclusion criteria on students and staff, including prior infection status. The numbers by age are described in Table S1. The ages for most of the individuals in the adult group (the staff) were not collected.

**Venous blood samples.** Blood samples were collected after prior consent by clinicians, nurses, and phlebotomists experienced in pediatric sampling using venipuncture and BD Vacutainer Gold Top serum separating gel tubes (SST; BD Diagnostics, USA). Samples were stored at room temperature during the collection at the school and shipped at room temperature, nonprocessed, to the laboratory on the same day, arriving within 8 h at the laboratory. After arrival at the laboratory, blood samples were stored in the fridge prior to separation of serum by centrifugation on the same day, and the serum was frozen prior to analysis (–20°C to −80°C).

**Oral fluids and extraction from Oracol swab.** The oral fluids were self collected on the same day as the paired venous blood, using the Oracol foam swab (Malvern Medical Developments [MMD], Worcester, United Kingdom), and shipped to the laboratory on the day of collection. The Oracol collects gingiva-crevicular fluid when brushing the gum line for 2 min, after which the swab is reinserted into a plastic container for transportation. Samples are stable for transport at ambient temperature (16, 29). Upon arrival, samples were stored in a fridge (4 to 8°C) and kept at room temperature during the process of booking into the laboratory database.

OF was extracted from the foam swab following the manufacturer’s recommended procedure (15) using 1 mL of an elution buffer (phosphate-buffered saline [PBS] containing 10% fetal calf serum, 250 μg/mL gentamicin, and 0.5 μg/mL amphotericin B [Fungizone]). The swab tube was centrifuged at 3,000 × *g* for 5 min in a benchtop centrifuge to remove cellular debris, the swab was removed and discarded, and 2 aliquots (a 500-μL aliquot for the capture/GICAP assays and the remainder for the RBD and total IgG assays) were created. Samples were stored at −20°C to −80°C prior to testing.

In accordance with our institutional risk assessment, samples were extracted inside a microbiological safety cabinet (MSC) in containment level 2 laboratories. No additional inactivation steps were undertaken. Initial assay steps (i.e., sample dilution onto assay plates and the first washing step) were performed inside an MSC.

### Oral fluid assays.

Three EIAs were developed in house, each based on the use of 96-well microplates, and comprised (i) an indirect EIA based on a solid-phase receptor-binding domain (RBD) antigen of S protein and horseradish peroxidase (HRP)-conjugated anti-human IgG probe (RBD25), (ii) IgG isotype capture antibody (GICAP) EIAs based on a solid-phase anti-human IgG with an HRP-conjugated viral NP probe, or (iii) GICAP EIAs based on a solid-phase anti-human IgG with an HRP-conjugated full-length viral spike protein probe.

### Laboratory analysis of oral fluids and sera.

**(i) IgG capture EIA NP and S.** Solid-phase wells (Nunc Immunomodule, U8 Maxisorp wells) were coated with 100 μL volumes of Affinipure rabbit anti-human v_°_ (Jackson ImmunoResearch, Ely, Cambridgeshire, UK) at 5 μg/mL in MicroImmune coating buffer for EIA with preservative (ClinTech, Guildford, UK). Coating was overnight at 2 to 8°C, followed by 3 h at 35 to 37°C. Wells were then washed with PBS Tween 20 and quenched with MicroImmune blocking solution (ClinTech, Guildford, UK) for 3 to 4 h at 37°C. Wells were aspirated and stored dry at 4°C in sealed pouches with desiccant until use. For both the NP and S capture EIAs, 100 μL of undiluted oral fluid was added to the wells and incubated for 60 ± 2 min at 37°C prior to washing and the addition of the conjugate. One hundred microliters of HRP-conjugated SARS-CoV-2 full-length spike glycoprotein (Native Antigen, Oxford, UK) or HRP-conjugated SARS-CoV-2 full-length nucleoprotein (Native Antigen, Oxford, UK) was added to the microwells for the S and NP assays, respectively. To mitigate potential issues with cross-reactivity in the NP capture assay, four recombinant seasonal coronavirus NP proteins (229E, NL63, OC43, and HKU1) were added to the SARS-CoV-2 NP conjugate at this step to act as blockers. After incubating for 60 ± 2 min at 37°C, the solid phase was again washed, 100 μL of substrate was added, the solution was incubated for 30 ± 2 min at 37°C, and the reaction was then stopped, and the optical density (OD) was measured at 450/630 nm.

**(ii) Indirect anti-RBD IgG EIA.** Oral fluids were analyzed with an in-house EIA established for analysis of sera (40, 44), which had been validated to guidelines by the International Council for Harmonization of Technical Requirements for Pharmaceuticals for Human Use (ICH) (45) in the early period of the pandemic and used for research purposes within our institute. It shows 93% sensitivity and 98% specificity, results that have been described in peer-reviewed publications (40, 46). Through checkerboard titration experiments, this indirect EIA was modified to allow analysis of oral fluids as follows: 96-well microtiter plates (Nunc, catalog no. 439454) were coated with recombinant SARS-CoV-2 RBD (Sino Biological Inc, 25 ng/well) incubated with sample at 1:50, and IgG in OF was detected using Biotin conjugated anti-human IgG(Fc) (eBiosciences, catalog no. 13-4998-83) followed by detection of the human IgG–anti-human IgG(Fc) complexes using streptavidin poly-HRP (ThermoScientific, catalog no. N200). During the optimization process, we established the OD of representative baseline samples (collected prepandemic, see Table S3 in the supplemental material). Using this information, we identified suitable negative material that could serve as a calibrator to determine test to negative (T/N) ratios as in the original, serum assay. The format of the OF assay was similar to that of the serum assay: a calibrator/negative sample (same specimen type as the clinical sample) was added in each quadrant of every plate and the T/N was calculated by dividing the geometric mean OD of each sample (analyzed in duplicate in separate halves of the plate) with the mean of the negative/calibrator. Each plate also contained a known positive, which was analyzed like a clinical sample in duplicate on each half of the plate.

**(iii) Determination of cutoff for all OF EIAs.** The cutoff for each assay was established separately in a stepwise manner initially based on the distribution of T/N values in negative samples (collected prior to and/or no later than April 2019) from individuals across the age groups (children, adults) by calculating S/CO + 3 times the standard deviation (3SD) of the S/CO values and T/N + 3SD for the GICAP EIAs and the RBD EIA, respectively.

The final cutoff value for OF samples was determined by age group (i.e., children and adults) from the serum-OF pairs using exploratory sensitivity versus specificity analysis (see Fig. S1 and Table S2 in the supplemental material), with the result from the commercial SARS-CoV-2 NP IgG serum EIA considered the true result. For each assay, the value that provided the highest possible sensitivity, while retaining the specificity minimum of 98% in both age groups, was chosen as the cutoff. These final cutoffs where then evaluated by AUC receiver operating characteristics (ROC) curve analysis shown in Fig. S1.

**(iv) Analysis with commercial EIA.** Contemporaneously collected serum samples were tested for SARS-CoV-2 IgG Abbott Architect SARS-CoV-2 IgG kit (reference no. 6R86 [detecting antibody to the NP of SARS-CoV-2]) following the manufacturer’s instruction using a cutoff above 0.8 to determine positivity. The use of this assay kit assay does not allow or involve dilutional steps of the serum samples.

At the time of sampling, May to July 2020, there were no commercially available assays that had gone through a rigorous development process for the measurement of SARS-CoV-2 antibody in this specimen type. We intended to compare the OF result with the results of an available and well-evaluated serum IgG measurement as a starting point for measuring SARS-CoV-2 serum IgG. The rationale for choosing the SARS-CoV-2 IgG Abbott Architect SARS-CoV-2 IgG kit (reference no. 6R86) as the gold standard was dependent on the fact that it had undergone a rigorous assessment process (39) and was approved for clinical use in the UK health system, and all sKIDs study blood results were released based on this test (i.e., the standard of care for this cohort).

Total IgG antibody concentrations were measured using the IgG human SimpleStep EIA kit (Abcam ab195215) according to the manufacturer’s instruction to determine the total (nonspecific, all subclasses) antibody level of IgG in the oral fluids only.

### Statistical analysis.

Sensitivity and specificity relative to those of the commercial SARS-CoV-2 NP IgG serum EIA (Abbott Architect) testing on serum were calculated with 95% exact confidence intervals. This was done at a range of cutoffs by ROC-curve analysis, which informed on the optimal cutoffs. The area under the ROC curve was also calculated with 95% confidence intervals as an overall measure of assay performance. Assays were compared visually using scatterplots with logged scaled axes and lines of best fit added, and Spearman’s correlation coefficient (rho) and its significance were calculated. This was also done to compare results to total IgG. Total IgG was compared between positive and negative results by Abbott on serum using Tobit regression.
