# Supplementary material for: SARS Antibody Testing in Children: Development of Oral Fluid Assays for IgG Measurements
Source: Microbiol Spectr. 2022 Jan 5;10(1):e00786-21. doi: 10.1128/spectrum.00786-21 (PMC8729769; doi:10.1128/spectrum.00786-21)

Supplement

Table S1: Summary of participant characteristics

	**Staff (Adults)**	**Children**	**Total**
**Number of participants**	**1253**	**746**	**1999**
**Period of collection**	**28th May – 10th July**		
**Male (%)**	**242 (19.31%)**	**377 (50.54%)**	**619 (30.97%)**
Sex unknown	30 (2.39%)	21 (2.82%)	51 (2.55%)
**Age at sampling median (min - max)**	**43 (22–71yrs)**	**8 (0–19yrs)**	**11 (0–71yrs)**
0 to 3 years	**Not applicable**	8 (1.07%)	8 (0.40%)
4 years		4 (1.55%)	4 (1.21%)
5 years		99 (13.27%)	99 (4.95%)
6 years		119 (15.95%)	119 (5.95%)
7 years		60 (8.04%)	60 (3.00%)
8 years		69 (9.25%)	69 (3.45%)
9 years		75 (10.05%)	75 (3.75%)
10 years		77 (10.32%)	77 (3.85%)
11 years		150 (20.11%)	150 (7.50%)
12 to 19 years		10 (1.34%)	10 (0.50%)
**Age unknown**	**750 (59.86%)**	**38 (5.09%)**	**787 (39.42%)**
Oral fluids with paired serum analysed in Abbot	1253 (100.00%)	746 (100.00%)	1999 (100.00%)
Paired OF with result in N capture	1251 (99.84%)	741 (99.33%)	1992 (99.65%)
Paired OF with result in S capture	1251 (99.84%)	741 (99.33%)	1992 (99.65%)
Paired OF with result in RBD indirect	1253 (100.00%)	746 (100.00%)	1999 (100.00%)
**OF with results for all three OF EIA's and data on total IgG**	**1251 (99.84%)**	**741 (99.33%)**	**1992 (99.65%)**

Table S2: Sensitivity and Specificity at a range of cut-offs based on serostatus determined by Abbot Architect (cut-off at 0.8)

	**RBD**					**NP**					**Spike**				
	**cut**	**n/N**	**Sens**	**n/N**	**Spec**	**cut**	**n/N**	**Sens**	**n/N**	**Spec**	**cut**	**n/N**	**Sens**	**n/N**	**Spec**
**sKIDS child**	2	66/92	0.72 (0.61-0.81)	633/654	0.97 (0.95-0.98)	0.7	79/91	0.87 (0.78-0.93)	582/650	0.90 (0.87-0.92)	0.7	79/91	0.87 (0.78-0.93)	540/650	0.83 (0.80-0.86)
	2.2	62/92	0.67 (0.57-0.77)	638/654	0.98 (0.96-0.99)	0.8	77/91	0.85 (0.76-0.91)	617/650	0.95 (0.93-0.97)	0.8	73/91	0.80 (0.71-0.88)	609/650	0.94 (0.92-0.95)
	2.4	58/92	0.63 (0.52-0.73)	643/654	0.98 (0.97-0.99)	0.9	76/91	0.84 (0.74-0.91)	631/650	0.97 (0.96-0.98)	0.9	72/91	0.79 (0.69-0.87)	636/650	0.98 (0.96-0.99)
	2.6	53/92	0.58 (0.47-0.68)	647/654	0.99 (0.98-1)	1	73/91	0.80 (0.71-0.88)	644/650	0.99 (0.98-1)	1	71/91	0.78 (0.68-0.86)	645/650	0.99 (0.98-1)
	2.8	51/92	0.55 (0.45-0.66)	648/654	0.99 (0.98-1)	1.1	71/91	0.78 (0.68-0.86)	645/650	0.99 (0.98-1)	1.1	66/91	0.73 (0.62-0.81)	648/650	1 (0.99-1)
	3	49/92	0.53 (0.43-0.64)	649/654	0.99 (0.98-1)	1.2	69/91	0.76 (0.66-0.84)	645/650	0.99 (0.98-1)	1.2	59/91	0.65 (0.54-0.75)	649/650	1 (0.99-1)
	3.2	47/92	0.51 (0.40-0.62)	650/654	0.99 (0.98-1)	1.3	65/91	0.71 (0.61-0.80)	646/650	0.99 (0.98-1)	1.3	52/91	0.57 (0.46-0.68)	650/650	1 (0.99-1)
**sKIDS adult**	2	137/196	0.70 (0.63-0.76)	977/1057	0.92 (0.91-0.94)	0.7	148/195	0.76 (0.69-0.82)	941/1056	0.89 (0.87-0.91)	0.7	151/195	0.77 (0.71-0.83)	896/1056	0.85 (0.83-0.87)
	2.2	134/196	0.68 (0.61-0.75)	995/1057	0.94 (0.93-0.96)	0.8	141/195	0.72 (0.66-0.79)	986/1056	0.93 (0.92-0.95)	0.8	137/195	0.70 (0.63-0.77)	978/1056	0.93 (0.91-0.94)
	2.4	127/196	0.65 (0.58-0.72)	1006/1057	0.95 (0.94-0.96)	0.9	140/195	0.72 (0.65-0.78)	1016/1056	0.96 (0.95-0.97)	0.9	128/195	0.66 (0.59-0.72)	1022/1056	0.97 (0.96-0.98)
	2.6	124/196	0.63 (0.56-0.70)	1019/1057	0.96 (0.95-0.97)	1	131/195	0.67 (0.60-0.74)	1041/1056	0.99 (0.98-0.99)	1	113/195	0.58 (0.51-0.65)	1044/1056	0.99 (0.98-0.99)
	2.8	122/196	0.62 (0.55-0.69)	1026/1057	0.97 (0.96-0.98)	1.1	125/195	0.64 (0.57-0.71)	1043/1056	0.99 (0.98-0.99)	1.1	99/195	0.51 (0.44-0.58)	1046/1056	0.99 (0.98-1)
	3	117/196	0.60 (0.53-0.67)	1035/1057	0.98 (0.97-0.99)	1.2	121/195	0.62 (0.55-0.69)	1044/1056	0.99 (0.98-0.99)	1.2	86/195	0.44 (0.37-0.51)	1047/1056	0.99 (0.98-1)
	3.2	109/196	0.56 (0.49-0.63)	1039/1057	0.98 (0.97-0.99)	1.3	119/195	0.61 (0.54-0.68)	1045/1056	0.99 (0.98-1)	1.3	78/195	0.40 (0.33-0.47)	1047/1056	0.99 (0.98-1)

[n = number (in a category); N = total number (of individuals); cut = cut-off level; Sens = sensitivity, Spec = Specificity]

Table S3: Pre-pandemic samples for OF validation:

Assay	Sample number	Age range (years)	Surveillance purpose	Description	Positives (%)
**RBD**	164	0-25	Mumps, Measles, Rubella (MMR)	Rash like illness (negative for MMR), collected 2019	3 / 164 (1.8%)
	325	2-16	Live attenuated influenza vaccine (LAIV) responses	Samples from vaccinated individuals (paired) for vaccine surveillance, collected 2014-2018	0 / 325 (0%)
	179	Adults (>/= 18 years)	Influenza-like illness	Pilot for national telephone survey with serological surveillance (pre- and post 2013/14 season)	1/179 (0.6%)
total	***668***				***4/668 (0.6%)***
**NP GICAP**	40	12-18	HAV transmission	Outbreak investigation in schools, collected 2018	1 / 40 (2.5%)
**Spike GICAP**	40	12-18	HAV transmission	Outbreak investigation in schools collected 2018	0 / 40 (0%)

Legend: The RBD and GICAP assays were developed and validated by separate research teams within the virology department, which determined access to separate pre-pandemic samples archived by these groups, which are detailed in the table.

Supplementary Figure S1: ROC analysis of the oral fluid assays. Receiver operating characteristic curves showing relationship of sensitivity and specificity when using the RBD EIA, NP GICAP and S-GICAP, respectively with OF samples collected from children (top row) and adults (bottom row), together with the area under the curve for each assay.

Supplementary Figure S2: Distribution of total IgG in OF by age group (i.e. children versus adults).

Figure S1: ROC Analysis

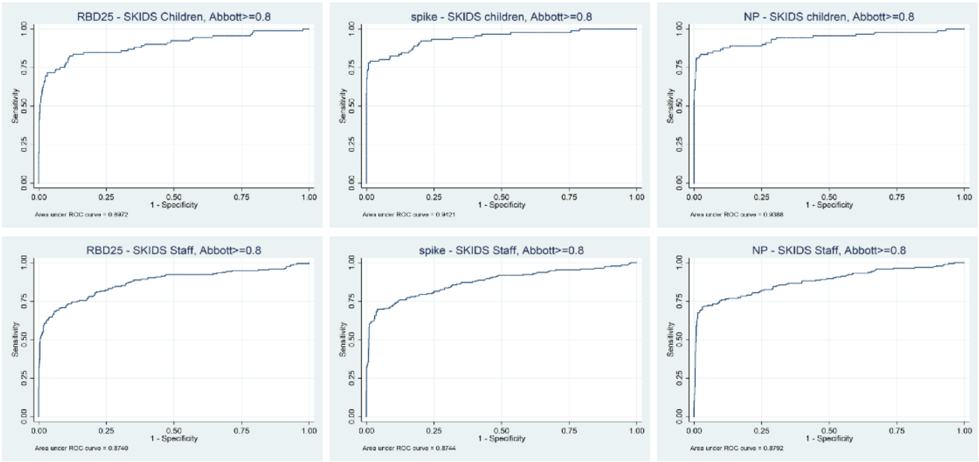

Area Under the Curve results (95% confidence Intervals)

Assay	RBD	Spike	NP
sKIDS child	0.897 (0.873-0.918)	0.942 (0.923-0.958)	0.939 (0.92-0.955)
sKIDS adult	0.874 (0.854-0.892)	0.874 (0.855-0.892)	0.879 (0.86-0.897)

ROC = receiver operating characteristic curve (vs Abbot at cut-off >=0.8)

Figure S2: Distribution of total IgG in OF by study group

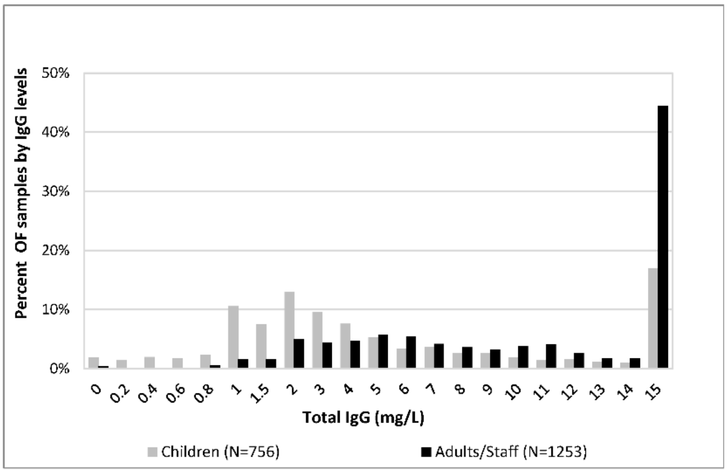

Supplement: SUPPLEMENTAL FILE 1 — Supplemental material. Download SPECTRUM00786-21_Supp_1_seq8.pdf, PDF file, 0.3 MB [file spectrum00786-21_supp_1_seq8.pdf]
